# The association between sex-related interleukin-6 gene polymorphisms and the risk for cerebral palsy

**DOI:** 10.1186/1742-2094-11-100

**Published:** 2014-06-06

**Authors:** Dan Bi, Mingjie Chen, Xiaoli Zhang, Honglian Wang, Lei Xia, Qing Shang, Tongchuan Li, Dengna Zhu, Klas Blomgren, Lin He, Xiaoyang Wang, Qinghe Xing, Changlian Zhu

**Affiliations:** 1 Department of Pediatrics, The Third Affiliated Hospital of Zhengzhou University, 7 Kangfu Street, 450052 Zhengzhou, China; 2 Children’s Hospital and Institutes of Biomedical Sciences, Fudan University, 130 Dong’an Road, 200032 Shanghai, China; 3 Department of Pediatrics, Zhengzhou Children’s Hospital, 255 Gangdu Street, 450053 Zhengzhou, China; 4 Department of Women’s and Children’s Health, Karolinska University Hospital, Q2:07, 17176 Stockholm, Sweden; 5 Center for Brain Repair and Rehabilitation, University of Gothenburg, 11 Medicinaregatan, SE-40530 Gothenburg, Sweden; 6 Bio-X Institutes, Key Laboratory for the Genetics of Developmental and Neuropsychiatric Disorders, Shanghai Jiaotong University, 1954 Huashan Road, 200030 Shanghai, China; 7 Perinatal Center, Institute of Neuroscience and Physiology, University of Gothenburg, 11 Medicinaregatan, 40530 Gothenburg, Sweden

**Keywords:** Cytokine, Inflammation, Periventricular leukomalacia, Single nucleotide polymorphisms

## Abstract

**Background:**

The relationship between genetic factors and the development of cerebral palsy (CP) has recently attracted much attention. Polymorphisms in the genes encoding proinflammatory cytokines have been shown to be associated with susceptibility to perinatal brain injury and development of CP. Interleukin-6 (IL-6) is a proinflammatory cytokine that plays a pivotal role in neonatal brain injury, but conflicting results have been reported regarding the association between *IL-6* single nucleotide polymorphisms (SNPs) and CP. The purpose of this study was to analyze *IL-6* gene polymorphisms and protein expression and to explore the role of *IL-6* in the Chinese CP population.

**Methods:**

A total of 753 healthy controls and 713 CP patients were studied to detect the presence of five SNPs (rs1800796, rs2069837, rs2066992, rs2069840, and rs10242595) in the *IL-6* locus. Of these, 77 healthy controls and 87 CP patients were selected for measurement of plasma IL-6 by Luminex assay. The SHEsis program was used to analyze the genotyping data. For all comparisons; multiple testing on each individual SNP was corrected by the SNPSpD program.

**Results:**

There were no differences in allele or genotype frequencies between the overall CP patients and controls among the five genetic polymorphisms. However, subgroup analysis found significant sex-related differences in allele and genotype frequencies. Differences were found between spastic CP and controls in males for rs2069837; between CP with periventricular leukomalacia and controls in males for rs1800796 and rs2066992; and between term CP and controls in males for rs2069837. Plasma IL-6 levels were higher in CP patients than in the controls, and this difference was more robust in full-term male spastic CP patients. Furthermore, the genotype has an effect on IL-6 synthesis.

**Conclusions:**

The influence of *IL-6* gene polymorphisms on IL-6 synthesis and the susceptibility to CP is related to sex and gestational age.

## Introduction

Cerebral palsy (CP) is a group of non-progressive motor impairment syndromes caused by lesions during early brain development that often include cognitive deficits and musculoskeletal dysfunction [1,2]. The prevalence of CP – around 2 to 3 cases per 1,000 live births – has been stable over the past 30 years [3]. CP has become the leading cause of childhood disability in both developing and developed countries [4,5], but the vast majority of the underlying pathogenic factors behind the development of CP are still unknown. Risk factors for CP have been categorized as prenatal, perinatal, and postnatal, but the majority of the risk factors – about 70% to 80% – occur prenatally [6]. Prenatal inflammation, such as intrauterine inflammation, is believed to be an important causal factor of adverse neurological outcomes [7], and an abnormal cytokine response has been suggested to be a common pathway for inducing brain injury [8].

Interleukin-6 (IL-6) is a pro-inflammatory cytokine that responds to intrauterine infection by inducing further increases in cytokine production [8]. Studies have shown that IL-6 is linked to clinical chorioamnionitis and preterm birth and that there is a three-fold increased risk for the development of periventricular leukomalacia (PVL) in preterm infants [9,10]. The *IL-6* single nucleotide polymorphism (SNP) rs1800795 (G-174C) was reported to be associated with disabling brain injury but not cognitive development in 148 children who were born at less than 32 weeks gestational age in the UK [9]. Additionally, a population-based case-control study including 334,333 infants showed that rs1800795 is a risk factor for CP among term and near-term infants who were born at more than 36 weeks gestational age in the US [11,12]. However, with comparable gestational age distributions in CP patients and control groups, conflicting results showed no association between CP and rs1800795 in 144 very preterm infants who were born at less than 32 weeks gestational age in Croatia [13]. The conflicting results might be based on differences in sample size or could indicate that the effect of *IL-6* gene function in the development of CP might be influenced by ethnicity or gestational age. Based on a replicate genetic study on SNPs from different regions of the *IL-6* gene, we have previously reported that the SNP rs2069837 is associated with male spastic CP in the Chinese Han population. To further confirm the risk factors associated with SNPs and to validate the regulation of IL-6 by the SNPs [10], we used a larger sample size to analyze five polymorphisms from different regions of the *IL-6* gene and combined this with a plasma IL-6 protein assay to evaluate the importance of IL-6 in the etiology of CP.

## Methods

### Subjects

The study population consisted of 713 CP patients (219 girls (30.7%) and 494 boys (69.3%) with a mean age ± SD of 19.1 ± 14.9 months) chosen from centers for CP rehabilitation in the Third Affiliated Hospital of Zhengzhou University and Zhengzhou Children’s Hospital from 1 July 2010 to 31 May 2012 (Table 1). Of these, 87 CP patients (54 boys (62.1%) and 33 girls (37.9%) with a mean age ± SD of 19.9 ± 14.1 months) were selected for the serum IL-6 assay (Table 2). The 753 healthy control participants (262 girls (34.8%) and 491 boys (65.2%) with a mean age ± SD of 19.6 ± 18.5 months) were chosen from the Child Healthcare Departments at the same hospitals during the same period. Of these healthy controls, 77 (63 boys (81.8%) and 14 girls (18.2%) with a mean age ± SD of 19.8 ± 12.4 months) were selected for the serum IL-6 assay. Blood samples were collected into tubes containing EDTA by skilled nurses on the second day of being hospitalized. Plasma was separated by centrifugation (1,500 × *g* for 15 min) at room temperature within 2 hours after being collected. DNA was obtained from the remaining blood components from the same sample. All the prepared samples were immediately stored at −70°C. All subjects were Han Chinese from the Henan Province, and written informed consent to participate in this study was provided by the children’s parents. Child neurologists diagnosed and classified the CP by clinical examination or by using medical records, including brain imaging, according to the guidelines proposed by the “Surveillance of CP in Europe” network [14]. Children in either the CP or control group with myopathy or metabolic anomalies were excluded from the analysis. For the serum IL-6 assay, the age of the selected children ranged from 5 months to 36 months. Children with cough, fever, acute respiratory illness, or any other any indications of infection within the past 3 months were excluded. Because of the genetic factors and familial factors that are associated with CP, we ensured that the controls had no familial relationships with the patients and did not have neurological symptoms. Approval for the study was obtained from the ethics committee of Zhengzhou University and the Medical Academy of Henan Province (201201002) in accordance with the Declaration of Helsinki.

**Table 1:** Sample description for gene polymorphism analysis

**Characteristic**	**CP cases**		**Control**	
	**Total (%)**	**M/F (n)**	**Total (%)**	**M/F (n)**
**Type of CP**				
Spastic CP	456 (64.0)	325/131	–	–
Non-spastic CP	257 (36.0)	169/88	–	–
Total	713 (100)	494/219	753 (100)	491/262
**Gestational age**				
Term (≥37 weeks)	496 (69.6)	338/158	742 (98.5)	481/261
Preterm (<37 weeks)	217 (30.4)	156/61	11 (1.5)	10/1
Total	713 (100)	494/219	753 (100)	491/262
**Birth weight**				
≥2,500 g	552 (77.4)	390/162	731 (97.1)	475/256
<2,500 g	161 (22.6)	104/57	22 (2.9)	16/6
Total	713 (100)	494/219	753 (100)	491/262
**Birth asphyxia**				
No asphyxia	484 (67.9)	326/158	741 (98.4)	482/259
Asphyxia	229 (32.1)	168/61	12 (1.6)	9/3
Total	713 (100)	494/219	753 (100)	491/262
**Complication**				
With PVL	75 (10.5)	56/19	–	–
Without PVL	638 (89.5)	438/200	–	–
With MR	274 (38.4)	185/89	–	–
Without MR	439 (61.6)	309/130	–	–
With HIE	342 (48.0)	245/97	–	–
Without HIE	371 (52.0)	249/122	–	–
**Maternal factors**				
PROM	88 (12.3)	62/26	14 (1.9)	11/3
No PROM	625 (87.7)	432/193	739 (98.1)	480/259
TPL	57 (8.0)	42/15	23 (3.1)	17/6
No TPL	656 (92.0)	452/204	730 (96.9)	474/256
PIH	35 (4.9)	23/12	14 (1.9)	12/2
No PIH	678 (95.1)	471/207	739 (98.1)	479/260

CP, cerebral palsy; M, male; F, female; PVL, periventricular leukomalacia; MR, mental retardation; HIE, hypoxic ischemic encephalopathy; PROM, premature rupture of membrane; TPL, threatened premature labor; PIH, pregnancy-induced hypertension.

**Table 2:** Sample description for cytokine quantification

**Characteristic**	**CP cases**		**Control**	
	**Total (%)**	**M/F (n)**	**Total (%)**	**M/F (n)**
**Type of CP**				
CP with spastic	58 (68.2)	35/23	–	–
CP without spastic	27 (31.8)	19/8	–	–
Total	85 (100)	54/31	75 (100)	62/13
**Gestational age**				
Term (≥37 weeks)	56 (65.9)	41/15	70 (93.3)	57/13
Preterm (<37 weeks)	29 (34.1)	13/16	5 (6.7)	5/0
Total	85 (100)	54/31	75 (100)	62/13
**Birth weight**				
≥2,500 g	61 (71.8)	43/18	71 (94.7)	59/12
<2,500 g	24 (28.2)	11/13	4 (5.3)	3/1
Total	85 (100)	54/31	75 (100)	62/13
**Birth asphyxia**				
No asphyxia	51 (60.0)	32/19	73 (97.3)	61/12
Asphyxia	34 (40.0)	22/12	2 (2.7)	1/1
Total	85 (100)	54/31	75 (100)	62/13
**Complication**				
With PVL	17 (20.0)	11/6	–	–
Without PVL	68 (80.0)	43/25	–	–
With MR	62 (72.9)	37/25	–	–
Without MR	23 (27.1)	17/6	–	–
With HIE	31 (36.5)	20/11	–	–
Without HIE	54 (63.5)	34/20	–	–
**Maternal factors**				
PROM	19 (22.4)	8/11	7 (9.3)	5/2
No PROM	66 (77.6)	46/20	68 (90.7)	57/11
TPL	20 (23.5)	15/5	15 (20.0)	11/4
No TPL	65 (76.5)	39/26	60 (80.0)	51/9
PIH	8 (9.4)	5/3	6 (8.0)	5/1
No PIH	77 (90.6)	49/28	69 (92.0)	57/12

CP, cerebral palsy; M, male; F, female; PVL, periventricular leukomalacia; MR, mental retardation; HIE, hypoxic ischemic encephalopathy; PROM, premature rupture of membrane; TPL, threatened premature labor; PIH, pregnancy-induced hypertension.

The database of medical records contains information on CP risk factors, such as birth asphyxia, symptoms concomitant with CP, such as mental retardation (MR), neonatal complications, such as PVL, and on hypoxic-ischemic encephalopathy (HIE). In addition, the records contain information on maternal factors such as premature rupture of membrane (PROM), pregnancy-induced hypertension (PIH), and threatening premature labor (TPL). Asphyxia was diagnosed using the criteria of the American Academy of Pediatrics and the American College of Obstetricians and Gynecologists, and newborns were included when at least three of the four criteria were met [15]. MR was identified based on a score of less than 70 on the Bayley Scales measurement of mental development index [16]. PVL was defined as parenchymal densities or lucencies around the ventricles during ultrasound examination of the head performed any time after 14 days post-birth [17], and this was further confirmed by MRI. Diagnosis of HIE required a combination of parameters that are indicative of metabolic acidosis within the first hours after birth. These include low umbilical cord blood pH (<7.0), a base deficit of over 12 mEq/L, and evidence of a need for respiratory support starting in the first minutes after birth with low Apgar scores at and beyond 5 min after birth. The diagnosis of PROM was based on pooling of amniotic fluid in the vagina, amniotic fluid ferning patterns, and a positive nitrazine test [18]. PIH was diagnosed and classified according to the criteria recommended by the American Congress of Obstetricians and Gynecologists, including a systolic blood pressure of 140 mmHg or higher or a diastolic blood pressure of 90 mmHg or higher on two occasions at least 6 h apart occurring after 20 weeks of gestation in a pregnant woman with previously normal blood pressure and without detectable urinary protein [19]. TPL was based on at least one of the following having occurred during pregnancy: the mother was admitted to the hospital prenatally with an episode of TPL, the mother received antenatal steroids or tocolytics, or a note was made on the delivery room chart that there was a history of TPL during the pregnancy [20].

### Polymorphism selection

A total of five SNPs (rs1800796, rs2069837, rs2066992, rs2069840, and rs10242595) of the *IL-6* gene whose minor allele frequencies in the Chinese Han population are more than 0.1 were selected from the dbSNP database (http://www.ncbi.nlm.nih.gov/SNP) and the hapmap human SNP database (http://www.hapmap.org). All of rs2069837, rs2066992 in the second intron, and rs2069840 in the third intron are tag SNPs. rs1800796 and rs10242595 are located in the upstream and downstream regions of the *IL-6* gene, respectively.

### Genotyping

Genomic DNA was prepared from venous blood using the AxyPrep Blood Genomic DNA Miniprep Kit (Axygen Biosciences, Union City, CA, USA) according to the manufacturer’s instructions. Probes and primers were designed by the SEQUENOM online tools (https://www.mysequenom.com) and the sequences are available upon request. After the amplification of polymorphism-spanning fragments by multiplex polymerase chain reaction (PCR), the genotyping was performed with the Sequenom MassARRAY SNP genotyping platform (Sequenom, San Diego, CA, USA). The person who analyzed the genotype results was blinded to the clinical data.

### IL-6 quantification

Blood samples were collected using EDTA as an anti-coagulant. The samples were centrifuged at 1,500 × *g* for 15 min at room temperature, and plasma was aliquoted and stored at −70°C. Before the assay, the frozen samples were thawed completely at room temperature, mixed well by vortexing, and centrifuged to remove precipitated material. IL-6 was assayed together with IL-8, IL-10, IL-17, TNF-α, or IFN-γ by using the Milliplex Human Cytokine/Chemokine Kits (Millipore, Billerica, MA, USA) according to the manufacturer’s instructions on a Luminex 200IS System (Luminex Corporation, Austin, TX, USA). Quality control was performed between the plates by using the two controls provided in the kit and representative samples from both CP patients and controls run on all the plates. The assay was completed on the same day by the same person. The plate variation was 6.94% in this assay. IL-6 levels were calculated using the Beadview software package (Upstate, Temecula, CA, USA) and expressed as pg/mL. The detection limit for IL-6 was 0.3 pg/mL.

### Statistical analysis

For gene analysis, the Hardy-Weinberg equilibrium (HWE) test was performed on the allele and genotype frequency analysis using the SHEsis online software platform (http://analysis.bio-x.cn). Linkage disequilibrium was measured using standardized D’, and the discrepancies in allele and genotype frequencies at single loci between patients and controls were compared using a Monte Carlo simulation strategy. The numbers of observations for each haplotype were compared using χ^2^ tests. The relative risk was approximated by the estimate of the odds ratio (OR). All ORs were adjusted for age and sex using logistic regression models. For each OR, a 95% confidence interval (CI) was calculated. For IL-6 cytokine analysis, the Student’s unpaired *t*-test was used. Data groups with unequal variances were analyzed using the Mann-Whitney U-test. All reported *P* values were two-tailed and statistical significance was set at *P* <0.05. The Statistical Package for the Social Sciences (SPSS version 19.0) and Graphpad Prism 6.0 software package (version 6.0 for Windows, Graphpad, La Jolla, CA, USA) were used for all statistical analyses. For all comparisons, multiple testing on each individual SNP was corrected by the SNPSpD program (http://gump.qimr.edu.au/general/daleN/matSpD/), which is based on the linkage disequilibrium information.

## Results

### Overall analysis

The genotypic distribution of the five selected SNPs in the controls was in HWE. There were no differences in allele or genotype frequencies between the total population of CP patients (n = 713) and total population of controls (n = 753) for any of the five genetic polymorphisms (Table 3). Computation of D’ indicated a strong linkage disequilibrium between these SNP markers (D’ >0.8) (Table 4). A haplotype analysis of all five SNPs between the CP and control subjects was performed (those with a haplotype frequency <0.03 were excluded from the analysis), and three common haplotypes were built. No differences in the haplotypes were found between CP cases and controls (data not shown).

**Table 3:** Allele and genotype distribution of the five selected SNPs in cerebral palsy patients and controls

	**Group**	**Allele frequency**		***P *** **value**	**Genotype frequency**			***P *** **value**	**Hardy-Weinberg equilibrium**
rs1800796		C	G		C/C	C/G	G/G		
	Case	977 (0.690)	439 (0.310)	0.256	347 (0.490)	283 (0.400)	78 (0.110)	0.300	0.081
	Control	992 (0.670)	488 (0.330)		334 (0.451)	324 (0.438)	82 (0.111)		0.797
rs2069837		A	G		A/A	A/G	G/G		
	Case	1138 (0.807)	272 (0.193)	0.062	469 (0.665)	200 (0.284)	36 (0.051)	0.144	0.018
	Control	1134 (0.779)	322 (0.221)		448 (0.615)	238 (0.327)	42 (0.058)		0.169
rs2066992		G	T		G/G	G/T	T/T		
	Case	441 (0.311)	977 (0.689)	0.389	77 (0.109)	287 (0.405)	345 (0.487)	0.316	0.140
	Control	483 (0.326)	999 (0.674)		77 (0.104)	329 (0.444)	335 (0.452)		0.775
rs2069840		C	G		C/C	C/G	G/G		
	Case	1300 (0.913)	124 (0.087)	0.503	591 (0.830)	118 (0.166)	3 (0.004)	0.787	0.258
	Control	1346 (0.906)	140 (0.094)		607 (0.817)	132 (0.178)	4 (0.005)		0.265
rs10242595		A	G		A/A	A/G	G/G		
	Case	1255 (0.891)	153 (0.109)	0.435	560 (0.795)	135 (0.192)	9 (0.013)	0.498	0.789
	Control	1279 (0.882)	171 (0.118)		569 (0.785)	141 (0.194)	15 (0.021)		0.079

**Table 4:** The linkage disequilibrium among the SNPs

**D’/r**^**2**^	**rs1800796**	**rs2069837**	**rs2066992**	**rs2069840**	**rs10242595**
rs1800796		0.884	0.981	0.971	0.899
rs2069837	0.463		0.903	0.998	0.777
rs2066992	0.948	0.497		0.986	0.912
rs2069840	0.203	0.026	0.211		0.949
rs10242595	0.228	0.025	0.235	0.704	

The standardized D’ values are shown above the diagonal, and the r^2^ values are shown below the diagonal.

### Subgroup analysis

Subgroup analysis of SNPs was performed according to sex, gestational age, birth weight, birth asphyxia, subtypes of CP, pregnancy complications, and maternal factors. Significant differences in allele frequencies were observed between CP patients (n = 494) and controls (n = 491) in males for rs2069837 (OR = 1.334, 95% CI = 1.073–1.659, *P* = 0.009, *P* = 0.027 after SNPSpD correction) (Table 5), but there were no differences in these SNP allele/genotype frequencies between CP patients and controls in females (data not shown).

**Table 5:** Allele and genotype distribution of the five selected SNPs in male cerebral palsy cases and controls

	**Group**	**Allele frequency**		***P *** **value**	**Genotype frequency**			***P *** **value**	**Hardy-Weinberg equilibrium**
rs1800796		C	G		C/C	C/G	G/G		
	Case	678 (0.690)	304 (0.310)	0.092	242 (0.493)	194 (0.395)	55 (0.112)	0.113	0.093
	Control	631 (0.655)	333 (0.345)		206 (0.427)	219 (0.454)	57 (0.118)		0.917
rs2069837		A	G		A/A	A/G	G/G		
	Case	790 (0.808)	188 (0.192)	**0.009**^**a**^	326 (0.667)	138 (0.282)	25 (0.051)	**0.041**	0.044
	Control	718 (0.759)	228 (0.241)		279 (0.590)	160 (0.338)	34 (0.072)		0.101
rs2066992		G	T		G/G	G/T	T/T		
	Case	303 (0.309)	677 (0.691)	0.119	53 (0.108)	197 (0.402)	240 (0.490)	0.159	0.193
	Control	330 (0.342)	634 (0.658)		55 (0.114)	220 (0.456)	207 (0.429)		0.764
rs2069840		C	G		C/C	C/G	G/G		
	Case	901 (0.914)	85 (0.086)	0.646	411 (0.834)	79 (0.160)	3 (0.006)	0.742	0.704
	Control	877 (0.908)	89 (0.092)		396 (0.820)	85 (0.176)	2 (0.004)		0.253
rs10242595		A	G		A/A	A/G	G/G		
	Case	870 (0.895)	102 (0.105)	0.304	391 (0.805)	88 (0.181)	7 (0.014)	0.578	0.426
	Control	838 (0.880)	114 (0.120)		372 (0.782)	94 (0.197)	10 (0.021)		0.168

^a^After the SNPSpD correction, the *P* value is 0.027.

Spastic CP was the main type of CP seen in this study population. Differences in allele frequencies were observed between spastic CP patients (n = 456) and controls (n = 753) for rs2069837, but the difference disappeared after SNPSpD correction (data not shown). Further analysis according to the sex of the CP patients revealed significant differences in allele and genotype frequencies between male spastic CP patients (n = 325) and controls (n = 491) for rs1800796, rs2069837, and rs2066992, but the differences for rs1800796 and rs2066992 disappeared after SNPSpD correction (Table 6), which was similar to our previous analysis. Haplotype analysis for rs1800796, rs2069837, and rs2066992 showed that the haplotypes “CAT” and “GGG” (rs1800796, rs2069837, and rs2066992) were strongly associated with male spastic CP patients (haplotypes with a frequency <0.03 were excluded from the analysis) (Table 7).

**Table 6:** Allele and genotype distributions of the SNPs in male spastic cerebral palsy patients and controls

	**Group**	**Allele frequency**		***P *** **value**	**Genotype frequency**			***P *** **value**	**Hardy-Weinberg equilibrium**
rs1800796		C	G		C/C	C/G	G/G		
	Spastic	458 (0.709)	188 (0.291)	**0.022**	169 (0.523)	120 (0.372)	34 (0.105)	**0.027**	0.073
	Non-spastic	220 (0.655)	116 (0.345)	0.995	73 (0.435)	74 (0.440)	21 (0.125)	0.944	0.739
	Control	631 (0.655)	333 (0.345)		206 (0.427)	219 (0.454)	57 (0.118)		0.917
rs2069837		A	G		A/A	A/G	G/G		
	Spastic	524 (0.816)	118 (0.184)	**0.007**^**a**^	220 (0.685)	84 (0.262)	17 (0.053)	**0.024**	0.022
	Non-spastic	266 (0.792)	70 (0.208)	0.223	106 (0.631)	54 (0.321)	8 (0.048)	0.457	0.740
	Control	718 (0.759)	228 (0.241)		279 (0.590)	160 (0.338)	34 (0.072)		0.101
rs2066992		G	T		G/G	G/T	T/T		
	Spastic	189 (0.293)	455 (0.707)	**0.040**	34 (0.106)	121 (0.376)	167 (0.519)	**0.041**	0.092
	Non-spastic	114 (0.339)	222 (0.661)	0.919	19 (0.113)	76 (0.452)	73 (0.435)	0.994	0.907
	Control	330 (0.342)	634 (0.658)		55 (0.114)	220 (0.456)	207 (0.429)		0.764
rs2069840		C	G		C/C	C/G	G/G		
	Spastic	599 (0.924)	49 (0.076)	0.245	276 (0.852)	47 (0.145)	1 (0.003)	0.489	0.498
	Non-spastic	302 (0.893)	36 (0.107)	0.440	135 (0.799)	32 (0.189)	2 (0.012)	0.496	0.947
	Control	877 (0.908)	89 (0.092)		396 (0.820)	85 (0.176)	2 (0.004)		0.253
rs10242595		A	G		A/A	A/G	G/G		
	Spastic	575 (0.901)	63 (0.099)	0.192	261 (0.818)	53 (0.166)	5 (0.016)	0.444	0.235
	Non-spastic	295 (0.883)	39 (0.117)	0.885	130 (0.778)	35 (0.210)	2 (0.012)	0.729	0.835
	Control	838 (0.880)	114 (0.120)		372 (0.782)	94 (0.197)	10 (0.021)		0.168

^a^After the SNPSpD correction, the *P* value is 0.021.

**Table 7:** Estimated IL-6 haplotype frequencies

**Haplotype**	**Case (frequency)**	**Control (frequency)**	***P *** **value**	**OR [95% CI]**
CAT	446.99 (0.705)	586.16 (0.634)	0.011^a^	1.331 [1.068–1.659]
GGG	113.50 (0.179)	202.19 (0.219)	0.039	0.764 [0.591–0.987]
Global results			0.037	

Loci chosen for haplotype analysis: rs1800796, rs2069837, and rs2066992.

Haplotype frequency <0.03 in both controls and cases have been dropped.

^a^After Bonferroni correction the *P* value is 0.033.

OR, odds ratio; CI, confidence interval.

PVL is common in preterm brain injury and is related to the development of CP. In this study, we found differences in genotype frequencies between CP patients with PVL (n = 56) and controls (n = 491) in males at rs1800796 and rs2066992 (Table 8). The haplotype analysis of all five analyzed SNPs did not show significant differences between CP patients with PVL and control subjects in either males or females.

**Table 8:** Allele and genotype distributions of the SNPs in male cerebral palsy (CP) patients with or without periventricular leukomalacia (PVL)

	**Group**	**Allele frequency**		***P *** **value**	**Genotype frequency**			***P *** **value**	**Hardy-Weinberg equilibrium**
rs1800796		C	G		C/C	C/G	G/G		
	CP + PVL	80 (0.714)	32 (0.286)	0.206	33 (0.589)	14 (0.250)	9 (0.161)	**0.014**^**b**^	0.004
	CP − PVL	598 (0.687)	272 (0.331)	0.136	209 (0.480)	180 (0.414)	46 (0.106)	0.272	0.437
	Control	631 (0.655)	333 (0.345)		206 (0.427)	219 (0.454)	57 (0.118)		0.917
rs2069837		A	G		A/A	A/G	G/G		
	CP + PVL	91 (0.812)	21 (0.188)	0.207	39 (0.696)	13 (0.232)	4 (0.071)	0.263	0.075
	CP − PVL	699 (0.807)	167 (0.193)	**0.013**^**a**^	287 (0.663)	125 (0.289)	21 (0.048)	0.057	0.131
	Control	718 (0.759)	228 (0.241)		279 (0.590)	160 (0.338)	34 (0.072)		0.101
rs2066992		G	T		G/G	G/T	T/T		
	CP + PVL	32 (0.286)	80 (0.714)	0.230	9 (0.161)	14 (0.250)	33 (0.589)	**0.013**^**c**^	0.004
	CP − PVL	271 (0.312)	597 (0.688)	0.171	44 (0.101)	183 (0.422)	207 (0.477)	0.348	0.705
	Control	330 (0.342)	634 (0.658)		55 (0.114)	220 (0.456)	207 (0.429)		0.764
rs2069840		C	G		C/C	C/G	G/G		
	CP + PVL	105 (0.938)	7 (0.062)	0.554	49 (0.875)	7 (0.125)	0 (0.000)	0.554	0.618
	CP − PVL	796 (0.911)	78 (0.890)	0.830	362 (0.828)	72 (0.165)	3 (0.007)	0.778	0.777
	Control	877 (0.908)	89 (0.092)		396 (0.820)	85 (0.176)	2 (0.004)		0.253
rs10242595		A	G		A/A	A/G	G/G		
	CP + PVL	99 (0.900)	11 (0.100)	0.543	45 (0.818)	9 (0.164)	1 (0.018)	0.821	0.500
	CP − PVL	771 (0.894)	91 (0.106)	0.341	346 (0.803)	79 (0.183)	6 (0.014)	0.603	0.542
	Control	838 (0.880)	114 (0.120)		372 (0.782)	94 (0.197)	10 (0.021)		0.168

^a^After the SNPSpD correction, the *P* value is 0.039.

^b^After the SNPSpD correction, the *P* value is 0.042.

^c^After the SNPSpD correction, the *P* value is 0.039.

According to gestational age at birth, the CP patients and controls were stratified as term or preterm CP cases. A difference in allele and genotype frequencies for the rs2069837 site was observed between term CP patients (n = 338) and term controls (n = 491) in males (Table 9), but no difference was found between preterm CP patients (n = 156) and preterm controls in males or between term and preterm CP patients in either males or females.

**Table 9:** Allele and genotype distributions of the SNPs in male term cerebral palsy patients and controls

	**Group**	**Allele frequency**		***P *** **value**	**Genotype frequency**			***P *** **value**	**Hardy-Weinberg equilibrium**
rs1800796		C	G		C/C	C/G	G/G		
	Case	463 (0.689)	209 (0.311)	0.137	162 (0.482)	139 (0.414)	35 (0.104)	0.282	0.525
	Control	617 (0.654)	327 (0.346)		20 (0.426)	215 (0.456)	56 (0.119)		0.897
rs2069837		A	G		A/A	A/G	G/G		
	Case	540 (0.811)	126 (0.189)	**0.012**^**a**^	220 (0.661)	100 (0.300)	13 (0.039)	**0.046**	0.699
	Control	702 (0.758)	224 (0.242)		272 (0.587)	158 (0.341)	33 (0.071)		0.134
rs2066992		G	T		G/G	G/T	T/T		
	Case	209 (0.312)	461 (0.688)	0.203	33 (0.099)	143 (0.427)	159 (0.475)	0.430	0.918
	Control	323 (0.342)	621 (0.658)		54 (0.114)	215 (0.456)	203 (0.430)		0.797
rs2069840		C	G		C/C	C/G	G/G		
	Case	610 (0.905)	64 (0.095)	0.838	276 (0.819)	58 (0.172)	3 (0.009)	0.701	0.980
	Control	859 (0.908)	87 (0.092)		388 (0.820)	83 (0.175)	2 (0.004)		0.271
rs10242595		A	G		A/A	A/G	G/G		
	Case	595 (0.891)	73 (0.109)	0.544	266 (0.796)	63 (0.189)	5 (0.015)	0.769	0.570
	Control	821 (0.881)	111 (0.119)		365 (0.783)	91 (0.195)	10 (0.021)		0.134

^a^After the SNPSpD correction, the *P* value is 0.036.

Differences in genotype frequencies were observed between CP patients with maternal PROM (n = 88) and controls (n = 753) for rs2066992 and rs1800796 (Table 10). Further analysis according to the sex of the CP patients showed differences between CP patients with maternal PROM (n = 88) and controls (n = 491) in males for rs2066992 and rs1800796, but the differences disappeared after SNPSpD correction.

**Table 10:** Allele and genotype distributions of the SNPs in patients with or without maternal premature rupture of membrane (PROM)

	**Group**	**Allele frequency**		***P *** **value**	**Genotype frequency**			***P *** **value**	**Hardy-Weinberg equilibrium**
rs1800796		C	G		C/C	C/G	G/G		
	CP + PROM	121 (0.695)	53 (0.305)	0.504	49 (0.563)	23 (0.264)	15 (0.172)	**0.006**^**a**^	0.001
	CP − PROM	856 (0.689)	386 (0.311)	0.292	298 (0.480)	260 (0.419)	63 (0.101)	0.560	0.572
	Control	992 (0.670)	488 (0.330)		334 (0.451)	324 (0.438)	82 (0.111)	0.011*^b^	0.797
rs2069837		A	G		A/A	A/G	G/G		
	CP + PROM	145 (0.824)	31 (0.176)	0.171	62 (0.705)	21 (0.239)	5 (0.057)	0.231	0.095
	CP − PROM	993 (0.805)	241 (0.195)	0.101	407 (0.660)	179 (0.290)	31 (0.050)	0.243	0.056
	Control	1134 (0.779)	322 (0.221)		448 (0.615)	238 (0.327)	42 (0.058)		0.169
rs2066992		G	T		G/G	G/T	T/T		
	CP + PROM	54 (0.307)	122 (0.693)	0.609	15 (0.170)	24 (0.273)	49 (0.557)	**0.005**^**c**^	0.001
	CP − PROM	387 (0.312)	855 (0.688)	0.425	62 (0.100)	263 (0.424)	296 (0.477)	0.663	0.750
	Control	483 (0.326)	999 (0.674)		77 (0.104)	329 (0.444)	335 (0.452)	0.012*^d^	0.775
rs2069840		C	G		C/C	C/G	G/G		
	CP + PROM	163 (0.926)	13 (0.0734	0.377	75 (0.852)	13 (0.148)	0 (0.000)	0.607	0.454
	CP − PROM	1137 (0.911)	111 (0.089)	0.635	516 (0.827)	105 (0.168)	3 (0.005)	0.889	0.339
	Control	1346 (0.906)	140 (0.094)		607 (0.817)	132 (0.178)	4 (0.005)		0.265
rs10242595		A	G		A/A	A/G	G/G		
	CP + PROM	153 (0.879)	21 (0.121)	0.915	68 (0.782)	17 (0.195)	2 (0.023)	0.990	0.459
	CP − PROM	1102 (0.893)	132 (0.107)	0.371	492 (0.797)	118 (0.191)	7 (0.011)	0.395	0.980
	Control	1279 (0.882)	171 (0.118)		569 (0.785)	141 (0.194)	15 (0.021)		0.079

*Comparison between total CP patients with maternal PROM and without PROM.

^a^After the SNPSpD correction, the *P* value is 0.0018.

^b^After the SNPSpD correction, the *P* value is 0.033.

^c^After the SNPSpD correction, the *P* value is 0.015.

^d^After the SNPSpD correction, the *P* value is 0.036.

### IL-6 quantification

**Figure 1:**
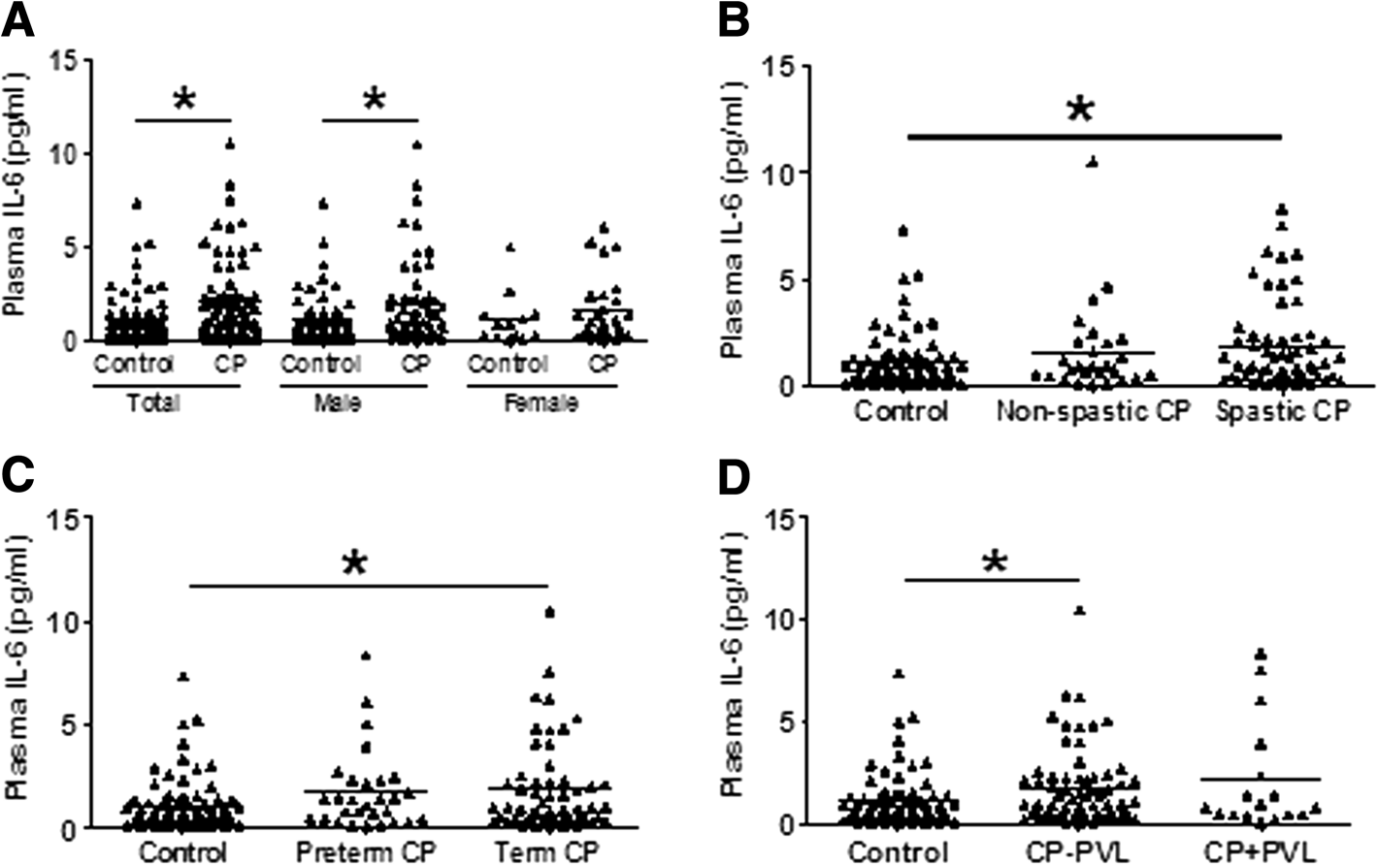
**Plasma IL-6 concentrations in CP patients and controls. (A)** Distribution of plasma IL-6 concentrations between total CP patients (85 patients) and total controls (75 participants) and between subgroups analyzed by sex. **(B)** Plasma IL-6 concentrations among spastic CP patients (58 patients), non-spastic CP patients (27 patients), and controls. **(C)** Plasma concentrations among term CP patients (56 patients), preterm CP patients (29 patients), and controls. **(D)** Plasma IL-6 concentrations among CP patients with PVL (17 patients), CP patients without PVL (68 patients), and controls. Each dot represents one patient and each bar represents the mean value. *P <0.05; Mann-Whitney U-test. CP, cerebral palsy; PVL, periventricular leukomalacia.

**Figure 2:**
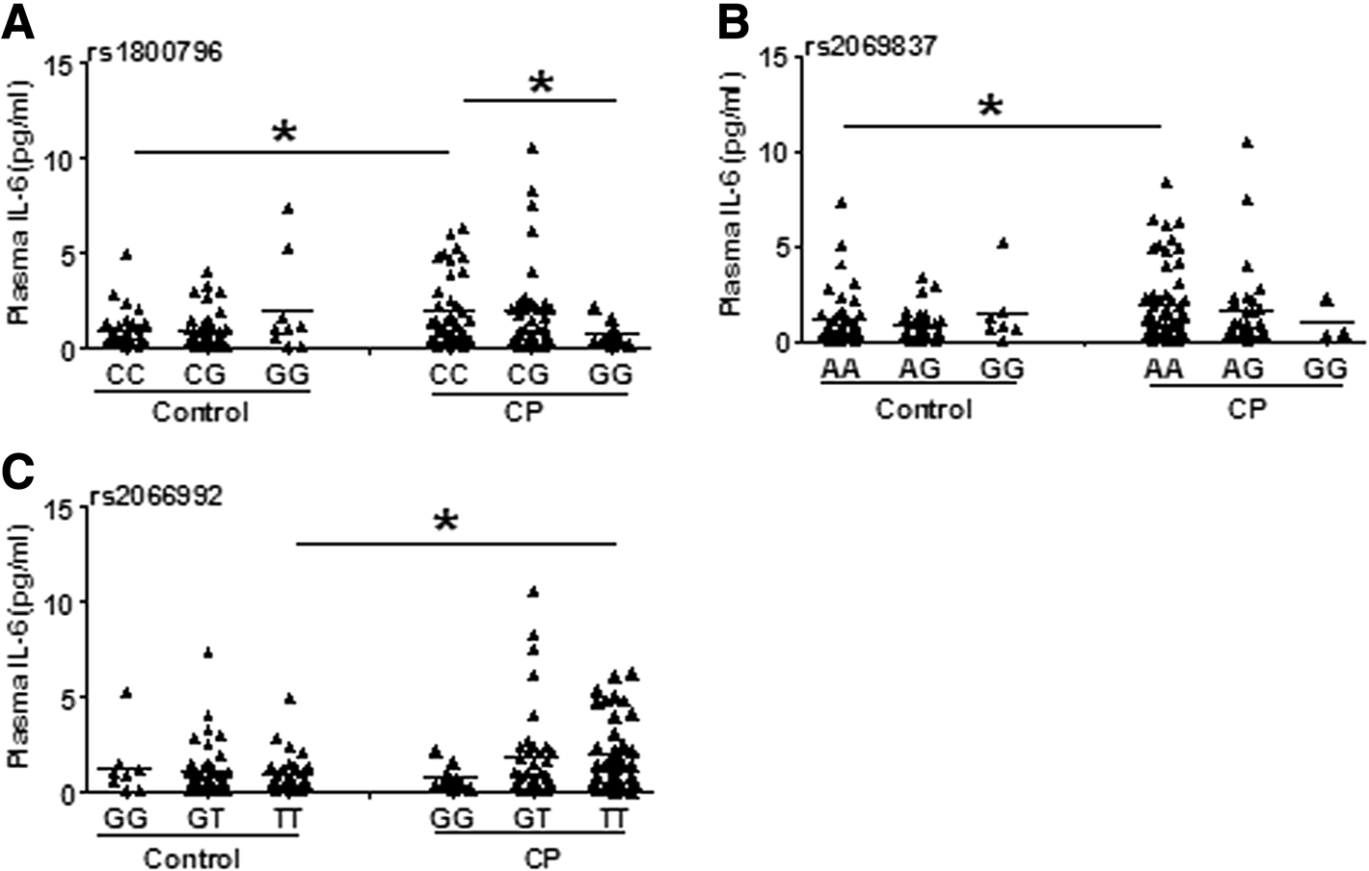
**Comparison of genotypes and plasma IL-6 levels in CP patients and controls. (A)** The comparison at rs1800796. **(B)** The comparison at rs2069837. **(C)** The comparison at rs2066992. Each dot represents one patient and each bar represents the mean value. *P <0.05; Mann-Whitney U-test. CP, cerebral palsy.

The mean plasma IL-6 concentration was higher in the CP patients than in the controls, and this difference was more pronounced in males (Figure 1A). Subgroup analysis showed that the IL-6 level was higher in the spastic CP group compared to the control group (Figure 1B). Furthermore, the IL-6 level in the CP patients was related to gestational age and a significant increase was seen in the full-term birth CP patients (Figure 1C). However, the IL-6 level was not changed compared to controls in the CP patients with PVL (Figure 1D). Further statistical analyses stratified by birth weight, birth asphyxia, MR, HIE, and maternal complications such as PIH, TPL, and PROM were also performed but no differences were found (data not shown). Furthermore, the plasma IL-6 levels were different between CP patients with genotypes CC and those with GG at rs1800796 (*P* = 0.033) (Figure 2A), and the plasma IL-6 levels of genotype CC at rs1800796 (Figure 2A), AA at rs2069837 (Figure 2B), and TT at rs2066992 (Figure 2C) in CP patients were higher than in their respective controls. Therefore, we have sufficient evidence to assume that there is a positive association of the IL-6 gene with the etiology of CP.

## Discussion

IL-6 is a proinflammatory cytokine that plays a key role in systemic inflammatory processes and has been identified as an important mediator of prenatal inflammation and CP. Clinical studies suggest that preterm neonates born to mothers with elevated IL-6 levels in the amniotic fluid are at increased risk for the subsequent development of PVL and CP [21], and CP patients show higher IL-6 levels in the cerebral spinal fluid [22]. Furthermore, infection has been shown to be an independent risk factor for spastic hemiplegia CP in infants born at term [23] and chorioamnionitis has been shown to be the most common cause of spastic quadriplegia CP [24]. In this study, we found that the plasma IL-6 levels were higher in CP patients than in controls and that IL-6 levels increased dramatically in spastic CP patients, which has not previously been reported. Overall, the findings from previous studies and our current work suggest that IL-6 is strongly associated with CP. Although we could not confirm whether the elevation of IL-6 levels is a recurrent or persistent issue that occurs after the event that led to the development of CP or if it is a transient effect during the late stage of CP; such increases might help to identify infants that could possibly benefit from later therapeutic interventions.

Genetic regulation of inflammation is an important biological risk factor that might contribute to the risk of developing CP [13]. The promoter region polymorphism rs1800795 (G-174C) in the *IL-6* gene has been associated with CP in populations from both Australia [8] and California [12]. The variant C allele of rs1800795 (G-174C) has been shown to markedly increase the risk of hemiplegic CP and quadriplegic CP in infants [8], and the CC genotype of the 174C/G variation is related to higher levels of IL-6 compared to the GG genotype [25].

Studies of *IL-6* polymorphisms have mainly focused on rs1800795 [8,12], but this polymorphism is absent in the Han Chinese population [26]. The SNPs rs1800796, rs2069837, and rs10242595 have all been reported to be associated with Alzheimer’s disease and ischemic stroke [27-30], and the rs2069840 SNP has been shown to be associated with systemic sclerosis [31]. The common haplotype tagged by the SNP rs1800796 (C-572G) has been shown to be associated with an increased risk of brain infarct in elderly subjects in a cardiovascular health study [32]. Even though these SNPs were associated with neurological disease in adults, so far there has been no evidence showing an association with CP. This study has, for the first time, shown that associations between *IL-6* polymorphisms and CP are related to sex, PVL, gestational age, and the clinical subtype of CP. The rs2069837 SNP is associated with male spastic CP patients born at full term, and the genotypes of rs1800796 and rs2066992 are associated with male CP patients with PVL.

The genotypes produced from rs1800796 and rs2066992 SNPs, however, showed deviation from HWE in CP patients with PVL. Deviations from HWE could point to a sampling bias, mistyping of genotypes, or spurious gene associations after population stratification [33]. We carefully re-analyzed the data based on the reasons mentioned above and found that all of the controls were in HWE. As Esser suggested, if only the genotype distribution of the patient group shows deviation from HWE, this might provide additional support for an association between the marker locus with the disease in question [33]. Therefore, our study showed that the genotypes produced from the two SNPs (rs1800796 and rs2066992) are more likely associated with male CP patients with PVL. On the other hand, we cannot exclude the potential problem of a small sample size (n = 56) after population stratification.

In addition to changes in SNP frequency, we also measured IL-6 levels directly and found that in CP patients the CC genotype of rs1800796 (C-572G) has an effect on serum levels of IL-6 compared to the GG genotype. This is similar to what has been seen in trauma patients carrying the −572 C allele or −572 G allele [34]. This suggests that the G allele of rs1800796 could reduce transcriptional activity of the IL-6 promoter. We also found that the plasma IL-6 levels of genotype CC at rs1800796, AA at rs2069837, and TT at rs2066992 in CP patients were higher than their respective controls. We speculate that the genetic variation might enhance or weaken the inflammatory response to some extent during the pathogenic process of CP, or it might influence the interaction between genetic factors and the environment. It seems that the C allele of rs1800796, the A allele of rs2069837, and the T allele of rs2066992 might be risk factors for CP. This must be further confirmed with even larger samples.

In this study population, 69.3% (494/713) of the CP patients were males, and this proportion is in accordance with reports from other nations [35-38]. This sex-related difference has also been noticed in *in vitro* and *in vivo* models of cerebral ischemia and neuronal injury [39]. However, even though sex hormones, such as estrogens, protect against brain injury [40], the circulating hormone levels cannot completely account for sex differences in the CP patients or in the developing brain [41]. This could be related to genetic background since it has been reported that female-derived neurons (XX) have a survival advantage compared with male-derived neurons (XY) [42].

PROM has been associated with increased risk for intrauterine infection and clinical chorioamnionitis [43,44], and it has been observed that infants born after PROM are at increased risk of white matter injury and subsequent CP diagnosis [45,46]. In our study, we found that the genotypes of SNPs rs1800796 and rs2066992 are associated with CP and that this association is more robust in CP patients with maternal PROM. This finding suggests that maternal infections can be an up-stream causative factor that contributes to CP.

In summary, our study suggests that IL-6 participates in the pathogenesis of CP in a sex-related manner and that *IL-6* gene polymorphisms are risk markers for male term infants and might be related to the development of PVL in male CP patients.

## Abbreviations

CI: Confidence interval; CP: Cerebral palsy; HIE: Hypoxic-ischemic encephalopathy; HWE: Hardy-Weinberg equilibrium; IL-6: Interleuken-6; MR: Mental retardation; OR: Odds ratio; PCR: Polymerase chain reaction; PIH: Pregnancy-induced hypertension; PROM: Premature rupture of membrane; PVL: Periventricular leukomalacia; SNPs: Single nucleotide polymorphisms; TPL: Threatening premature labor.

## Competing interests

The authors declare that they have no competing interests.

## Authors’ contributions

CZ and QX conceived the project. DB, TL, QS, DZ, XZ, and LX provided samples. HW, MC, and DB performed most of the experiments. DB, HW, and MC analyzed the data. DZ and QS provided support when collecting samples. XW, LH, and KB provided support for the project and valuable discussions. DB, CZ, and QX wrote the paper. All authors read and made comments on the manuscript during its drafting. All authors read and approved the final manuscript.
